# Beyond Common Arrhythmias: Discovering Brugada Syndrome in a Patient With Nonspecific Symptoms

**DOI:** 10.7759/cureus.103952

**Published:** 2026-02-20

**Authors:** Noel George Cherian, Srinivasan Ravindranath

**Affiliations:** 1 General Practice, Thumbay University Hospital, Ajman, ARE; 2 Cardiology, HealthHub Clinic, Dubai, ARE

**Keywords:** brugada ecg pattern, brugada syndrome, inherited arrhythmias syndrome, scn5a mutation, transthoracic echocardiography (tte)

## Abstract

Brugada syndrome is an inherited cardiac channelopathy associated with life-threatening ventricular arrhythmias and sudden cardiac death and is often detected incidentally. We report a 48-year-old Filipino man who presented with persistent upper respiratory symptoms and intermittent atypical chest pain, with a history of recurrent syncope. Physical examination and cardiac biomarkers were normal. Electrocardiography revealed a spontaneous type 1 Brugada pattern in leads V2-V3. Echocardiography showed mild left ventricular hypertrophy, and exercise stress testing was negative for ischemia. Based on clinical and electrocardiographic findings, implantable cardioverter-defibrillator placement was advised. This case emphasizes the importance of recognizing Brugada syndrome in patients presenting with nonspecific symptoms to enable timely diagnosis and appropriate risk stratification.

## Introduction

Brugada syndrome (BrS) is a unique familial cardiac channelopathy, and it was initially recognized as a distinct clinical entity in 1992 by brothers Pedro and Josep Brugada. The Brugada brothers first became aware of such a case in 1986, when a three-year-old boy from Poland presented with several bouts of syncope. His electrocardiogram (ECG) exhibited ST-segment elevation in leads V1-V3. His sister demonstrated an analogous clinical and electrocardiographic presentation and succumbed at two years of age despite treatment with a pacemaker and amiodarone. Subsequently, many cases with similar features were observed, thus defining a previously unrecognized clinical condition [1,2].

Cardiac channelopathies are a group of illnesses characterised by defective ion channel activity in the heart, resulting in varied clinical symptoms. BrS is often transmitted via an autosomal dominant inheritance pattern. The initial genetic mutations associated with the condition were identified in 1998 within the SCN5A gene located on chromosome 3p21, which encodes the α-subunit of the cardiac sodium channel [2]. It predominantly affects young males and demonstrates higher prevalence in Southeast Asian populations, with an estimated global prevalence of approximately three to five per 10,000 individuals. It has three characteristic ECG patterns. The type 1 pattern is defined by a coved ST-segment elevation exceeding 2 mm followed by T-wave inversion, whereas type 2 demonstrates a saddleback morphology with ST-segment elevation greater than 2 mm. Type 3 is characterized by a saddleback configuration with less pronounced ST-segment elevation of under 2 mm [3,4]. These distinctive ECG changes, consisting of ST-segment elevation with subsequent T-wave inversion, are typically appreciated in the right precordial leads (V1-V3) [5]. Differential diagnoses of a right precordial ST-segment elevation include acute myocardial ischemia, early repolarization, arrhythmogenic right ventricular cardiomyopathy, pericarditis, and electrolyte disturbances, all of which must be excluded prior to confirming the diagnosis [6]. The following case report of a 48-year-old Filipino male with BrS illustrates the key steps in diagnosis, risk stratification, and management, while also highlighting important clinical considerations in patient care.

## Case presentation

**Figure 1:**
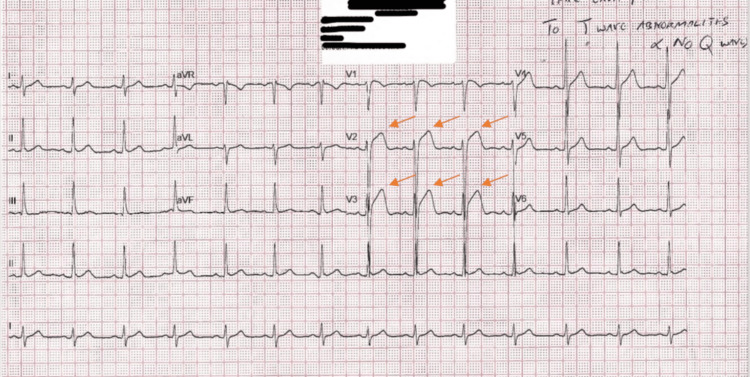
Electrocardiogram revealed coved ST segment elevations in V2 & V3 without reciprocal changes. Findings were highly suggestive of a type 1 Brugada pattern.

A 48-year-old Filipino male patient presented to the Internal Medicine outpatient department, complaining of a dry cough and rhinorrhea persisting for more than two weeks, accompanied by occasional chest pain of similar duration. The chest pain was atypical and localized over the left precordial region with no radiation. The most recent chest pain episode occurred two days prior to presentation, and the patient rated the pain as 3/10 in intensity. He had been taking over-the-counter medications for symptoms of the common cold and cough, but reported no significant relief. On detailed review, there was a history of mild dyspnea and multiple episodes of syncope at a younger age. His recent syncopal episode was five years ago, which had no apparent trigger. He was known to have hyperlipidemia, which was well controlled through dietary measures. There was no personal or family history of cardiac disease; however, his father had type 2 diabetes mellitus. The patient was vitally stable, except for a mildly elevated blood pressure, and the physical examination was unremarkable with palpable and symmetrical peripheral pulses bilaterally. Cardiovascular examination revealed normal S1 and S2 heart sounds, with no audible murmurs or pericardial rubs, and respiratory examination showed clear lung fields with normal breath sounds and equal air entry on both sides. The complete blood count, serial measurements of troponin I, creatine kinase-MB, and C-reactive protein were all within normal limits. A standard 12-lead ECG revealed coved ST-segment elevations mostly seen in leads V2 & V3 without reciprocal changes. Findings were highly suggestive of a spontaneous type 1 Brugada pattern (Figure 1). 

**Figure 2:**
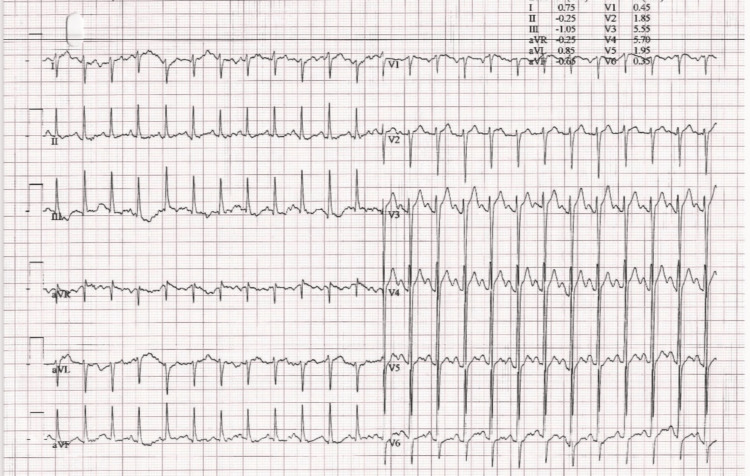
Exercise stress test stage 3. Overall test was negative for inducible ischemia.

**Figure 3:**
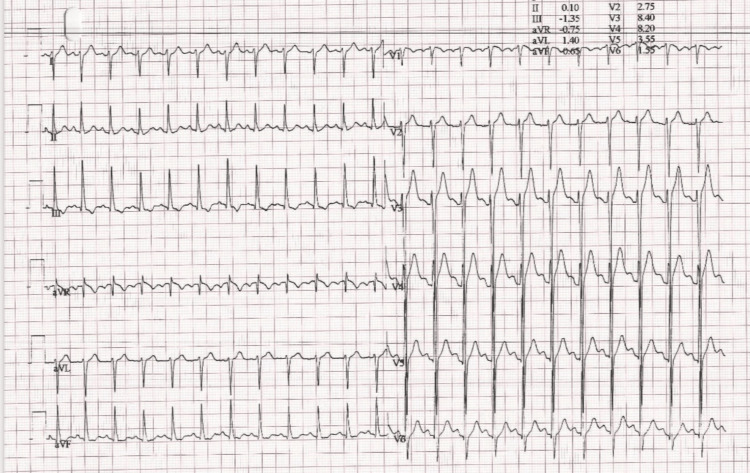
Exercise stress test recovery. Overall test was negative for inducible ischemia

The patient was referred to a cardiologist for further testing, which included a transthoracic echocardiography and stress test. The exercise stress test was negative for inducible ischemia (Figures 2, 3).

Echocardiography findings were unremarkable except for mild left ventricular hypertrophy. Genetic testing was not performed due to its limited availability in our clinic. Family screening and counseling were initiated for the patient. In view of the patient’s history of multiple syncopal episodes, implantation of a permanent cardioverter-defibrillator (ICD) was advised to monitor for and prevent potentially fatal arrhythmias. In addition, lifestyle modifications were recommended as part of his long-term management. The internal medicine specialist prescribed symptomatic treatment for the cough and rhinitis and advised the patient to return for follow-up if his symptoms worsened or failed to improve.

## Discussion

BrS is an inherited cardiac channelopathy, typically transmitted in an autosomal dominant manner, and is linked to a heightened risk of sudden cardiac death [7]. BrS is implicated in up to 20% of sudden cardiac deaths worldwide among patients with hearts that are structurally normal. The estimated global prevalence of the Brugada ECG pattern is approximately 0.4%, with significant regional and sex-based differences. However, the presence of this ECG pattern alone does not consistently correlate with increased risk of all-cause or cardiac death [8]. The prevalence of BrS varies worldwide, with the highest rates observed in Southeast Asia (3.7 per 1,000), followed sequentially by the Middle East, South Asia, East Asia, Europe, and North America. The global prevalence of the confirmed syndrome is expected to be 0.5 per 1000 [4]. Regarding the ethnicity of our patient case, we had a 48-year-old Filipino male patient, which was very much consistent with the usual BrS ethnicity predominance according to the widespread literature.

**Figure 4:**
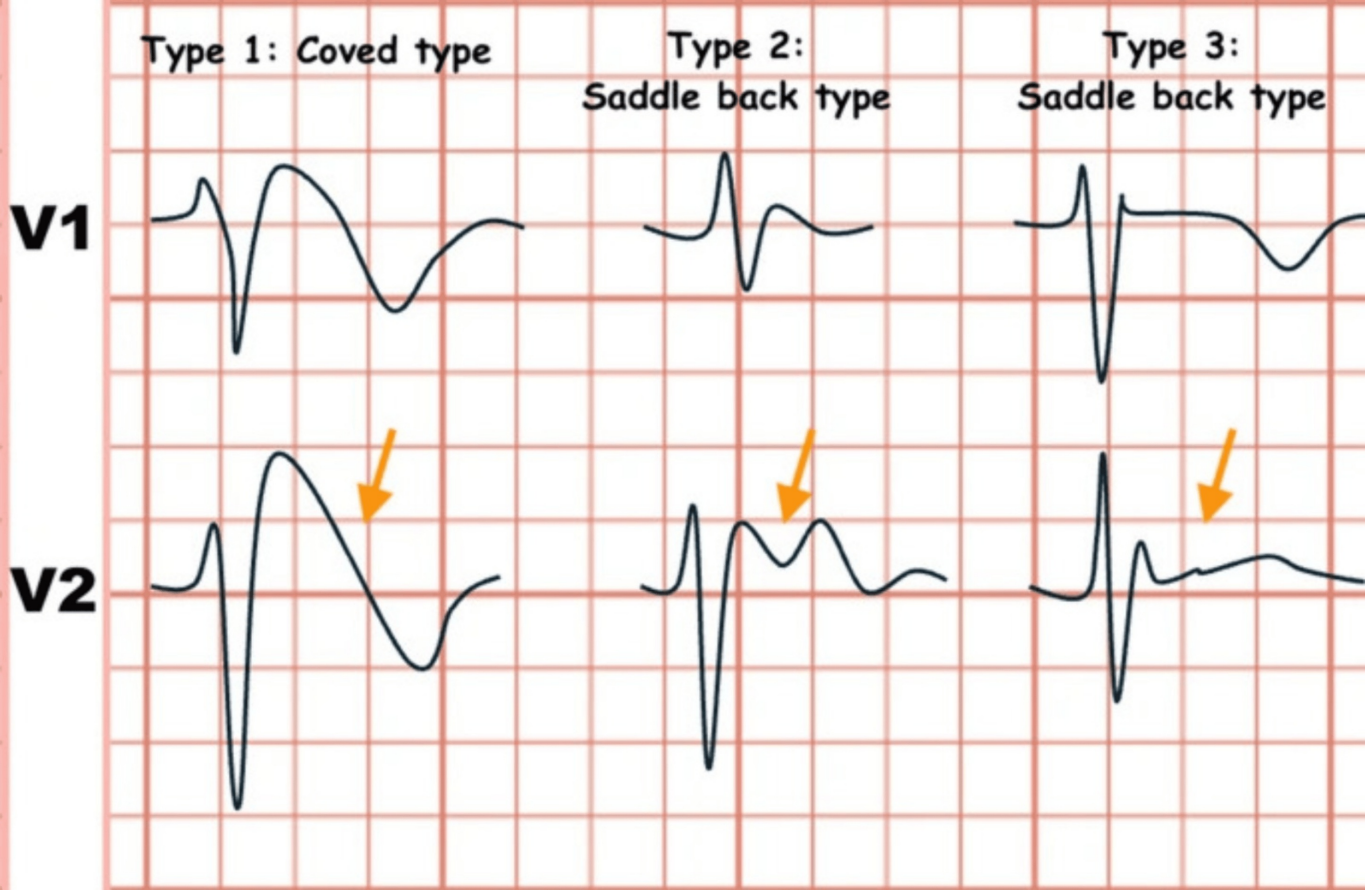
Brugada electrocardiogram patterns Image is under Creative Commons Attribution 4.0 International License [13].

The primary ion channel abnormality in BrS is a loss of function of the cardiac sodium channel, most commonly due to SCN5A gene mutations, leading to a diminished inward sodium current during depolarization [9]. Abnormalities in cardiac ion channel currents generate a transmural voltage gradient across the right ventricular outflow tract, producing the characteristic Brugada ECG pattern [10]. The Brugada ECG pattern is classified into three distinct repolarization morphologies observed in the right precordial leads (Figure 4). Among these, the type 1 Brugada ECG pattern is diagnostic and is characterized by a coved ST-segment elevation greater than 2 mm (0.2 mV) followed by T-wave inversion. In patients with suspected concealed Brugada syndrome, a diagnostic type 1 pattern can be elicited using a sodium channel-blocking challenge even when a spontaneous pattern is absent. The type 2 pattern displays a saddleback morphology with an ST-segment elevation of ≥2 mm, a trough elevation of ≥1 mm, and a positive or biphasic T wave. By comparison, the type 3 pattern is defined by less marked ST-segment elevation (<1 mm) with either a saddleback or coved appearance [11-13]. In our case, the ECG pattern did involve “coved-type” ST-elevations in V2 and V3 without any T-wave changes and absence of Q-waves.

It is safe to visualize that the Brugada ECG pattern, in our case, was relatively benign in view of the history of recurrent syncope. There has been a local case report involving a certain febrile illness trigger, where a 47-year-old presented to the hospital after experiencing a transient episode of syncope lasting several minutes. In the days preceding admission, the patient had developed a febrile illness accompanied by cough and sore throat. He reported three prior episodes of transient loss of consciousness over the past five years, predominantly occurring during periods of fever. Family history was significant for sudden cardiac death in a sibling at 41 years of age. Initial ECG demonstrated features characteristic of BrS, in addition to prolongation of the PR and corrected QT intervals [14]. This was quite similar to our case except for the positive family history and additional ECG changes. It is possible for a patient not to have a familial history of cardiac abnormalities or sudden death [15]. In addition to the presence of a characteristic Brugada ECG pattern, the diagnosis of BrS requires fulfillment of one or more established clinical criteria. These include reported ventricular fibrillation, a family history of sudden cardiac death before the age of 45 years, non-sustained polymorphic ventricular tachycardia, the presence of a Brugada ECG pattern in a first-degree relative, inducible ventricular tachyarrhythmias on electrophysiological study, unexplained syncope suggestive of an arrhythmic etiology, and episodes of nocturnal agonal respiration [16]. An electrophysiological study may be considered in selected patients for further risk assessment, although its prognostic utility remains debated. Our patient had multiple episodes of syncope. The Brugada ECG pattern represents a relatively benign entity that is most commonly unmasked in the setting of fever and is associated with favorable clinical outcomes following treatment of the precipitating condition. Meanwhile, our case had not identified any specific triggers such as fever, drug use, or electrolyte abnormalities. Based on a study analysis, patients demonstrating a Brugada ECG pattern alone do not require implantable cardioverter-defibrillator placement or antiarrhythmic therapy. Resolution of the ECG abnormalities is generally observed following appropriate management of the underlying trigger. As these individuals are typically asymptomatic, the condition often remains unrecognized and is most frequently identified incidentally during electrocardiographic evaluation [11].

The current mainstay symptomatic treatment is implantable cardioverter-defibrillators (ICD). ICD implantation was advised in this case in accordance with the second AHA consensus conference. Individuals presenting with syncope, seizure-like episodes, or nocturnal agonal respiration require comprehensive evaluation to exclude non-cardiac causes, after which ICD implantation may be indicated based on overall risk stratification [17-19]. However, there is always a slight chance of complications occurring post-ICD implantation, notably arrhythmic events and inappropriate shocks. To counteract this, strategies do exist to minimize inappropriate shock rates, including device selection and programming, medication, catheter ablation, and remote monitoring. Overall, ICDs are useful in reducing the risk of sudden cardiac death, but many patients with an ICD will receive an inappropriate shock. Understanding strategies to prevent inappropriate shocks is crucial to improving the care of patients with ICDs [20].

## Conclusions

This case illustrates the diagnostic challenge of Brugada syndrome presenting alongside seemingly benign symptoms. The patient’s high-prevalence origin, coupled with a spontaneous type 1 ECG pattern, in the context of recurrent syncope, highlights a high-risk profile that warranted implantable cardioverter-defibrillator placement. Accurate differentiation between true Brugada syndrome and benign Brugada ECG patterns is essential for appropriate risk stratification and to avoid unnecessary interventions. Early recognition, specialist referral, and patient education, including family screening and counseling, remain central to optimizing outcomes. Ongoing follow-up is also critical to monitor arrhythmic risk and ensure long-term management.
